# Leicester Health Advisory Committee

**Published:** 1918-08-10

**Authors:** 


### THE LEICESTER HEALTH ADVISORY COMMITTEE.

In our issue of June 15 we gave a brief description of the work of the Leicester Public Medical Service. We pointed out that the medical men of Leicester had organised an admirable service on a self-supporting and voluntary basis for the treatment of the uninsured poor of the town. Leicester has now taken another step forward. A conference of representatives of the medical profession and of organisations in Leicester specially interested in public health met there recently under the presidency of the Mayor (Alderman J. North). The conference decided that a committee of thirty representatives of local public bodies and health organisations should be formed. The committee will consider in an advisory capacity matters connected with the health of the town. It will, of course, have no executive powers and will not in the slightest degree interfere with the autonomy of the various public bodies and institutions. The Leicester Public Medical Service took the initial steps which led to the conference and it will be strongly represented upon the committee.

In all the schemes which have been formulated for the constitution of a Public Medical Service under a Ministry of Health stress has been laid, and rightly so, upon the formation of advisory committees to assist local authorities in their public health work. Leicester is once more showing how much can be done along the lines a Ministry of Health should follow, without waiting for special legislation, by medical men when they are well organised. The committee is bound to have a most important and beneficial influence upon public health work in Leicester. For the first time the medical profession as a whole will have an opportunity of exercising one of its most important functions—that of advising the community upon the best steps to be taken to prevent the occurrence of disease and to deal with it when it arises. Hitherto such advice has been given by individual medical men as officials of public bodies or in a private capacity. Such advice can be, and, only too frequently, has been ignored by public authorities without arousing any opposition on the part of the general public. It will be more difficult to ignore with impunity the advice of a committee of representatives of the whole of the medical profession and of organisations interested in medical work in a locality. Although such a committee cannot have executive powers, one may be quite sure that it will be impossible for any public body to act against its recommendations unless it can show very good cause for doing so. As one of the speakers at the conference stated, few medical men have the time or the inclination to fight for seats upon a popularly elected body, and, therefore, the medical profession has never exercised in the direction of public health work the influence to which its expert knowledge entitles it. The formation of this Health Advisory Committee will now enable it in Leicester to put this expert knowledge at the disposal of the community with advantage to the public and to itself.

